# Discrete Modeling Approach for Cluster-Based Excess Gibbs-Energy of Molecular Liquids

**DOI:** 10.1021/acs.iecr.3c03084

**Published:** 2023-11-07

**Authors:** Christoph Mayer, Thomas Wallek

**Affiliations:** Institute of Chemical Engineering and Environmental Technology, Graz University of Technology, Graz, 8010, Austria

## Abstract

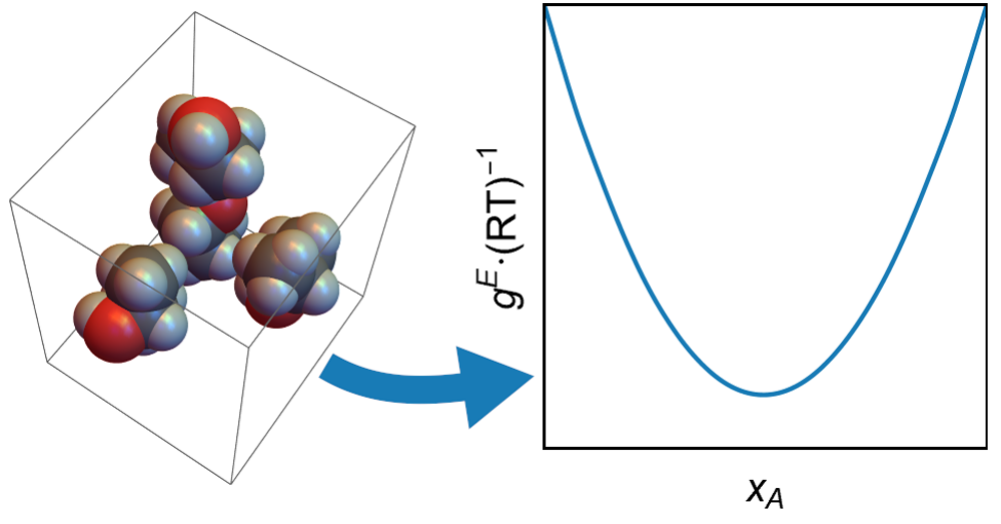

The excess Gibbs-energy of a two-component liquid molecular mixture is modeled based on discrete clusters of molecules. These clusters preserve the three-dimensional geometric information about local molecule neighborhoods that inform the interaction energies of the clusters. In terms of a discrete Markov-chain, the clusters are used to hypothetically construct the mixture using sequential insertion steps. Each insertion step and, therefore, cluster is assigned a probability of occurring in an equilibrium system that is determined via the constrained minimization of the Helmholtz free energy. For this, informational Shannon entropy based on these probabilities is used synonymously with thermodynamic entropy. A first approach for coupling the model to real molecules is introduced in the form of a molecular sampling algorithm, which utilizes a force-field approach to determine the energetic interactions within a cluster. An exemplary application to four mixtures shows promising results regarding the description of a variety of excess Gibbs-energy curves, including the ability to distinguish between structural isomers.

## Introduction

Activity coefficients and excess Gibbs-energy (*g*^E^) models on which they are based are important tools to estimate properties and phase equilibria of fluid-phase mixtures in the course of process engineering calculations using commercial simulation tools as they account for deviations of mixture behavior from the random mixing model. In particular, against the background of growing shares of biogenic components in value chains, complex molecules pose a challenge in terms of their rigorous thermodynamic description with such models. Increasingly nonspherical shapes and a large number of functional groups in the structures of such molecules make it challenging to apply established *g*^E^ models for reliable estimations of the macroscopic thermodynamic properties of mixtures.

Such thermodynamic models typically attribute the deviation of a mixture from random mixing to molecular shapes and sizes and to intermolecular interactions between the chemical components. Consequently, the cooperative effects due to such interactions prevent single molecules from being treated as statistically independent subsystems in the sense of statistical thermodynamics. As a remedy for this, various simplifications at different levels of sophistication have been proposed in model development to date.

Approaches which assume the contact pairs within a mixture to be statistically independent resulted in the so-called quasichemical models which are based on the ideas of Guggenheim^1−3^ and are still being developed today and frequently used in chemical engineering applications. Among the typical representatives of this type are UNIQUAC,^4^ UNIFAC,^5,6^ GEQUAC,^7^ MOQUAC,^8^ COSMO-RS respectively COSMOTHERM,^9−13^ and PAC-MAC.^14−17^ All of these models have in common that they assume interacting surface segments to be decoupled and only use their number densities to calculate mutual interactions. Consequently, the three-dimensional information on molecular configurations gets lost, which can only be overcome by *a posteriori* considering second-order effects that recover local surface correlations.^18^

A less simplistic approach to consider cooperative effects is to use molecular clusters as a modeling basis. Compared to pair approximations, clusters intrinsically preserve the three-dimensional geometric constraints affecting the intermolecular interactions between surface segments by considering only geometrically realistic cluster states. This *a priori* preservation of geometric information can be seen as an essential peculiarity of cluster-based approaches. As a recent example, the binary quantum cluster equilibrium (bQCE) theory^19−24^ has been developed to estimate the activity coefficients of liquid mixtures. Other cluster-based approaches use a Flory–Huggins term, e.g., combined with a force field-based molecular sampling algorithm^25^ or even go beyond the original Flory–Huggins approximations, like the reference interaction site model (RISM)^26,27^ and the lattice cluster theory (LCT).^28,29^ Probably the most stringent treatment of clusters is found in the so-called cluster variation methods (CVM)^30−32^ which have been well-developed for considering clusters of moderate size^33^ and have been successfully applied to the construction of phase diagrams for alloys.^34^ One limitation of CVM models is their complexity and extensive number of variables with increasing cluster size, requiring special algorithms to solve the CVM equations,^35,36^ which is the reason why CVM approaches have not been developed toward activity coefficient models for chemical engineering applications.

As an approximation to CVM methods, Vinograd et al. introduced the Markov-chain theory for the hypothetical, sequential construction of a lattice^37,38^ with the aim to predict two-dimensional crystal structures. As a unique feature, this substantially different approach uses Shannon information describing the sequential construction process of the system with clusters as a synonym for thermodynamic entropy. In previous papers, the two-dimensional Vinograd approach was further developed to three-dimensional simple cubic lattices of spherical molecules and dice systems.^39−43^

As a continuation of our work so far, in this paper, the previously developed dice model for two-component systems is extended to a lattice-free binary system of real molecules of arbitrary shapes, including a proof of concept in the form of a first application to selected two-component systems. The paper is structured as follows:

First, the thermodynamic model is explained, presenting the modeling concept based on clusters, the sequential construction of the system as a discrete Markov chain, and the set of variables involved. With these, the thermodynamic functions internal energy, entropy, and Helmholtz free energy are derived, together with the constraints that are applied to minimize the latter and that are essential for reducing the degrees of freedom in the course of this minimization. All of this results in the final system of equations for the model.

Second, a force-field-based method for determining the cluster energies is developed, as a first approach to link the model with data from real molecular systems. These cluster energies represent the substance-specific input parameters for the model.

Third, results of applying the model are discussed, beginning with an assessment of its boundary cases including model consistency in the limiting case of spherical molecules, demixing, a checkerboard-like arrangement of molecules, and random mixing. As a proof of concept, the model is then applied to describe nonrandom mixtures considering cooperative effects as they occur in real molecular systems.

Finally, the work is summarized, conclusions are drawn, and an outlook on the further development of the model is given.

## Model

The thermodynamic model addresses two-component systems of molecular liquids in the Gibbsian variable space of entropy, *S*, internal energy, *U*, and molecule numbers, *N*_*i*_. Its key concept is based on molecular clusters that are picked out from the overall system and act as representative subsystems. As is common with activity coefficient models, the volume is not taken into account, specifically because the underlying cluster model does not include the system volume as a function of cluster volumes in its current implementation.

The discrete modeling concept applied below is based on the rigorous modeling of entropy in terms of Shannon information, where molecule clusters are chosen as the smallest unit of modeling. The entropy of the system is determined from its hypothetical sequential construction in terms of a discrete Markov-chain. Complementarily, to account for cooperative effects, the internal energy of the system is calculated from the internal energies of the individual clusters, which are determined additively from the interactions of the nearest neighbors inside a cluster.

### Clusters as a Basis for Modeling

**Figure 1:**
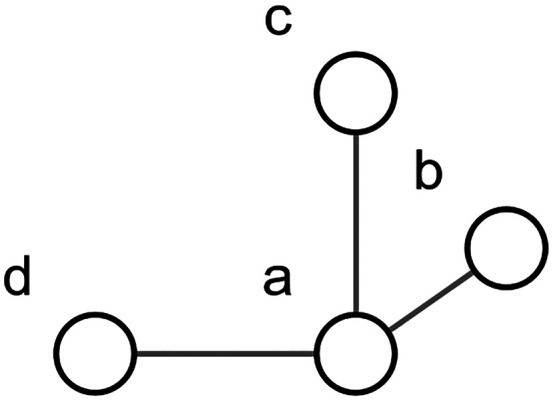
Graphical representation of the used cluster shape with cluster site labels ‘a’, ‘b’, ‘c’, and ‘d’. Each cluster site is occupied by one molecule.

The model uses clusters as its core building blocks. These clusters all share the same general shape and consist of four molecules placed into a 3-dimensional space. Figure 1 illustrates the cluster shape, where the spherical placeholders mark positions where the molecules are located.

**Figure 2:**
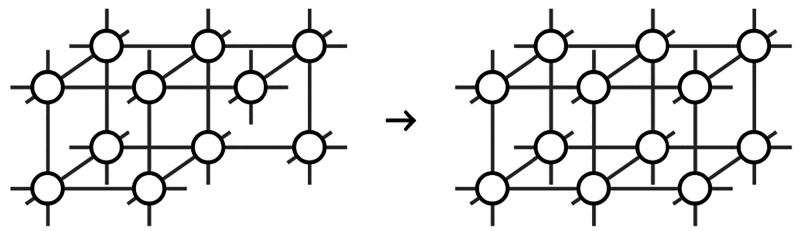
Hypothetical, sequential construction of the system as a discrete Markov chain by inserting a new molecule, where the three nearest neighbors are already present as a result of previous insertion steps; idealized representation as lattice system.

The individual cluster sites are labeled ‘a’, ‘b’, ‘c’, and ‘d’. The molecule at site ‘a’ occupies a central location and forms the origin of a Cartesian coordinate system. The other three molecules are placed along the axes of the coordinate system. In the figure, the axes are represented as lines connecting the sites. While the sites in Figure 1 are represented by spheres, the molecules which are inhabiting these sites can have arbitrary shapes and are not restricted to spherical molecules. The shape of the cluster is based on a previously established model that uses dice-like molecules in a simple cubic lattice. There, the system is hypothetically constructed by inserting a new molecule into a given neighborhood of already existing molecules from previous insertion steps.^43^ One such insertion is illustrated in Figure 2.

A newly inserted molecule of this sequential construction is only exposed to half of its nearest neighbors, i.e., half of the coordination number at the time of insertion, as the other nearest neighbors in the final lattice have not yet been placed. Based on these conditions, the cluster energies can be determined. Knowledge of the cluster energies is required for the calculation of the internal energy, assuming that the internal energy of a cluster can be calculated by the sum of the energies of nearest neighbor pairs. The chosen cluster shape contains three nearest neighbor pairs, each involving the central molecule ‘a’. These involve “a-b”, “a-c”, and “a-d”. A detailed description of how cluster energies are currently determined is given in the sampling section of this paper.

It should be noted that, although Figure 2 shows an idealized representation as a lattice system, the model does not make use of any lattice-related properties or constraints; only the cluster construction is done along the three Cartesian spatial axes, resulting in a coordination number of six. However, this restriction to only six nearest neighbors is a gross simplification of the actual conditions in a liquid where other models account for ten nearest neighbors,^4^ or, actually, even more are to be expected.^44^

### Molecular States

The molecules that are placed at the cluster sites can reside in several states. These are determined by (i) the component type, (ii) the molecular orientation, and (iii) the distances of molecules from their neighbors. Although the distance is not a molecular property, it is necessary to consider it in the context of clusters. As the distance is always a property of pairs of molecules, it is, however, convenient for modeling to assign this property to single molecules. For this purpose, the distance is split into two parts: The part that is assigned to the central molecule at site ‘a’ is universal to all of its neighbors, and the differences between the distances of the three pairs of a cluster have to be provided by the partial distance assigned to the neighboring sites ‘b’, ‘c’, and ‘d’. This allocation of molecular distances will be explained later in the course of presenting the sampling approach.

Within the model, the first distinction, the component type, is treated separately in the nomenclature. The two component types of the binary mixture are labeled *A* and *B*. The set consisting of both components is .
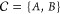
1

The other two distinguishing features of a molecule state, namely, the orientation and distance to its neighbors, are grouped together into geometric states. In the current implementation of the model, each component type is assigned the same number of geometric states, *m*, disregarding different molecular complexities of the components in terms of shapes and interaction sites.

### Possible Clusters and Cluster States

The hypothetical sequential construction allows for the newly inserted molecule to be in any molecular state. Since the neighboring molecules are the result of previous insertion steps, they as well can reside in all possible states. Therefore, possible clusters comprise all combinations of the molecular states. Mathematically, this means that all permutations with repetitions of the possible molecular states at all four cluster sites must be considered during modeling. This can be expressed as a well-known urn problem where the molecular states are represented by differently colored balls: For each of the four cluster sites, a ball is drawn from the urn; between each draw, all balls are returned to the urn, allowing multiple sites to have the same states. The resulting number of permutations, and thus clusters, is determined by raising the number of molecular states by the number of cluster sites, which is four. The resulting (2·*m*)^4^ clusters form the population of possible cluster states on which the model is based.

### Cluster Probabilities

The considerations above provide a list of possible clusters with assigned energy values. The model has to determine the probability distribution of these clusters in thermodynamic equilibrium, which is achieved via minimization of the Helmholtz energy that is modeled using these probabilities. The cluster probabilities are written as *p*_*abcd*_^*ijkl*^, where the subscripts indicate the component types at each cluster site and the superscripts indicate the geometric state. The site ‘a’ corresponds to the indices *a* and *i*, site ‘b’ to *b* and *j*, site ‘c’ to *c* and *k*, and site ‘d’ corresponds to *d* and *l*. With these definitions, the internal energy and entropy of the system are derived in the following.

### Internal Energy of the System

The potential internal energy of the system, *u*, is calculated from two properties of each cluster, i.e., the potential interaction energy of the cluster, *RTe*_*abcd*_^*ijkl*^, and the probability of the cluster to occur in the system, *p*_*abcd*_^*ijkl*^.
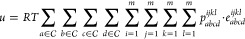
2Here, *R* represents the universal gas constant, *T* represents the temperature, and *m* is the number of geometric states per component. According to eq 2, the internal energy of the system is simply the weighted sum of the cluster interaction energies; the weights are the cluster probabilities. The sum over all possible discrete states is expressed with separate sums for each of the eight indices (*a*, *b*, *c*, *d*, *i*, *j*, *k*, and *l*) to keep a consistent nomenclature with upcoming equations.

### Entropy of the System

The entropy of the system represents the core of the model in this paper. It is equivalent to the Shannon information describing the sequential cluster construction process, illustrated in Figure 2. The general shape of the entropy function, as defined in eq 3, is the same as that of the previously published dice model.^43^
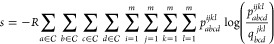
3

In addition to the already established cluster probabilities, *p*_*abcd*_^*ijkl*^, new probabilities, *q*_*bcd*_^*jkl*^, are introduced. These represent the probabilities of a certain neighborhood that is defined by the molecules located at sites ‘b’, ‘c’, and ‘d’ according to Figure 1. By the insertion of a new molecule into this neighborhood, the cluster is generated. The neighborhood probabilities can be determined from the cluster probabilities using
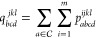
4

### Helmholtz Free Energy and Gibbs Free Enthalpy of the System

With the entropy, eq 3, and the internal energy, eq 2, the Helmholtz free energy of the system, *a*, is given by the thermodynamic relation
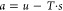
5

The variables of the free energy are the cluster energies *e*_*abcd*_^*ijkl*^ and the cluster probabilities *p*_*abcd*_^*ijkl*^. The cluster energies are substance-specific input parameters for the model and will be provided by a molecular sampling algorithm explained in detail further below. The cluster probabilities are model variables to be determined by the minimization of the free energy as a function of these probabilities to describe a system in thermodynamic equilibrium.

Due to the aforementioned fact that the volume is not taken into account, the enthalpy of the system equals the internal energy. Consequently, the Gibbs free enthalpy, *g*, that will be used for model assessment in the form of its excess quantity, *g*^E^, equals the Helmholtz free energy, which is evident from
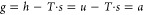
6

### Constraints Applied to Minimize the Helmholtz Free Energy

For constrained minimization of the Helmholtz free energy to determine the cluster probabilities of the system in thermodynamic equilibrium, further assumptions concerning the system are introduced in the form of constraints. These reduce the degrees of freedom in the course of this minimization.

### Grouping Cluster into Classes

As the modeling approach in this paper is an extension of a previous model based on spherical molecules, constraints from that model can be utilized.^42^ For this, the clusters are first grouped into classes based on the component type at each cluster site. This means that all clusters that share the same values for the indices *a*, *b*, *c*, and *d* are grouped together into a class. The probability of such a class is denoted *p*_*abcd*_. With this class definition, reflecting permutations with repetitions, there are 2^4^ = 16 class probabilities.

The remaining information that is needed to describe a cluster is the geometric state. Its probability contribution to the cluster probability can be expressed as a conditional probability based on the classes. Later in this paper, these conditional probabilities are approximated by distribution functions to reduce the number of free variables in the system; therefore, they are labeled as *dist*_*abcd*_^*ijkl*^. The connection between the different probabilities resulting from the class definition are given in
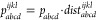
7

### Class Approximations

The establishment of classes opens the opportunity to introduce constraints for the classes. Up to this point, the equations used offer an exact description of the model assumptions. The model, however, contains many degrees of freedom. Therefore, approximations are introduced to reduce these and add additional information to the model. With this in mind, the class probabilities are approximated by three pairs of molecules (*p*_*ab*_, *p*_*ac*_, *p*_*ad*_), with the only dependency that they share a common molecule at site ‘a’.
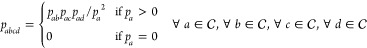
8The simplifying assumption of otherwise independent pairs allows for the simple multiplication of the probabilities of the molecule pairs, which is evident from eq 8. The denominator corrects the fact that the central molecule is counted multiple times.

With the introduction of this approximation, there is no geometric information on the cluster left in the class probabilities aside from the information of which molecule resides at the central site ‘a’. Classes with the same composition of neighbors share the same value for their respective class probability.

### Global Compositions

As the model is desired to yield the excess Gibbs free enthalpy, *g*^E^, at a specified global composition (i.e., the global molar fractions, *x*_*A*_, *x*_*B*_), the latter needs to be introduced into the system of equations. This is easily accomplished, since the global composition is identical to the probability of a component type to be present at an arbitrary cluster site, as given in
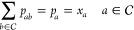
9

Here, the law of total probability is used, analogous to the calculation of the neighborhood probabilities from the cluster probabilities. Adding up the probabilities of all possible ways to construct a pair, where one of its molecules is fixed, results in the probability of that fixed molecule being found at an arbitrary position in the system. The latter is identical to the global composition, since eq 9 only differentiates between the component types.
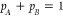
10

Along with the fact that the sum of all probabilities must equal one, see eq 10, all 16 class probabilities can be calculated using only the global composition of component *A*, *p*_*A*_, and one pair probability, *p*_*AB*_, as explained in a previous paper.^42^

It is worth mentioning that the pair probability *p*_*AB*_ is equivalent to the local composition parameter ξ introduced by Guggenheim and McGlashan^2^ in their quasichemical model.

### Probability Distributions for Cluster Classes

Having defined constraints for the classes, the conditional probabilities *dist*_*abcd*_^*ijkl*^ of eq 7 which are based on these classes, require further constraints as well. These probabilities describe the probability distribution of the clusters of class members, originating from the geometric states of the molecules.

As an approximation, probability distribution functions are now introduced to deal with the potentially large number of free variables during optimization. To achieve a suitable balance between a small number of variables and model flexibility, one distribution function is used per class. It is important that the chosen distribution function can take various forms: In particular, certain boundary cases give insight into the required shape flexibility of the probability distributions. The two boundary cases to be described are (i) systems with negligible energetic interactions, *u* = 0, and (ii) systems with dominating energetic interactions, *s* = 0.

In the first case, by reducing eq 5 to *a* = 0 – *T*·*s*, the minimization of the Helmholtz free energy reduces to a maximization of the entropy, resulting in a uniform distribution.

In the second case, by reducing eq 5 to *a* = *u* – *T*·0, the minimization of the Helmholtz free energy can be reduced to a minimization of the internal energy. Then, the resulting distributions maximize the probability of the clusters with the smallest energy while making the probabilities of clusters with large energies as small as possible. This requires a left-leaning probability distribution. A more detailed analysis of these boundary cases can be found in the results section.

The necessity for the distribution function to support both of these boundary cases has already significantly limited the number of possible distribution functions. Some common mathematical distributions, e.g., the beta-binomial distribution, would be able to satisfy these constraints. However, an additional property should be taken into consideration, which makes such common functions unsuitable: Within each class, the key distinguishing property of individual clusters is the cluster energy. Without taking it into account, the cluster probabilities would have to be labeled with categorical data, and the desired approximation would not be possible using distribution functions. Therefore, a custom distribution function is used that includes the energies, which enables clusters with a similar energy value to have a similar probability, as well.

As the first development step, a distribution function with one parameter, α_*abcd*_, is chosen. For the model, the defining characteristic of a cluster is its energy. Consequently, the latter is used to shape the distribution function for the conditional probabilities as follows:
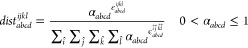
11The distribution function, eq 11, is in essence just the distribution parameter raised to the power of the cluster energy, with a normalization step to ensure that the sum of the conditional distribution probabilities for each class equals one. For reasons of numerical stability, it is beneficial to first shift the cluster energies to positive numbers. This step does not influence the shape of the distribution and is illustrated in
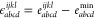
12

**Figure 3:**
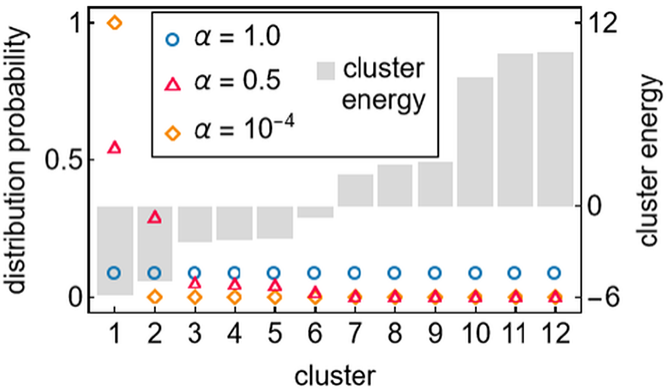
Illustration of the flexibility of the distribution function, eq 11, to describe different shapes with varying parameters α. This fictitious example uses 12 randomly chosen cluster energies, *e*_*abcd*_^*ijkl*^. The clusters are sorted in ascending order by their energy values.

The variables ϵ_*abcd*_^*ijkl*^ are the shifted positive energies, and *e*_*abcd*_^min^ is the minimal cluster energy for the class *abcd*. A fictitious example distribution is illustrated in Figure 3, to show how the distribution function relates to the cluster energies for different values of the distribution parameter α_*abcd*_.

Each of the 16 classes is thus described by using a one parameter distribution function. With these 16 distribution parameters, together with the pair probability discussed above, *p*_*AB*_, the number of model variables totals 17.

### Final System of Equations

The overall model is as follows:
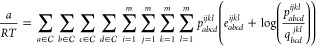
13a
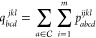
13b
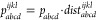
13c
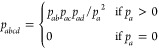
13d
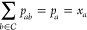
13e
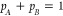
13f
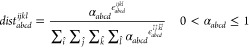
13g
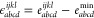
13h

The 17 system variables to be determined by the constrained minimization of *a*/*RT* include the pair probability, *p*_*AB*_, and the 16 distribution parameters, α_*abcd*_. The input parameters for the model comprise the temperature, *T*, the molar fraction, *x*_*a*_, and the cluster energies, *e*_*abcd*_^*ijkl*^. The numerical solution was accomplished using the L-BFGS-B algorithm as implemented in the SciPy Python package.^45−47^ Thermodynamic consistency of the model was proven numerically using a Gibbs–Helmholtz equation, as outlined in the Supporting Information, which contains the used strategies for generating initial values for numerical optimization as well.

## Sampling Approach for Cluster Energies

The interface between the model and real molecular mixtures is the list of cluster energies. These capture the energetic interactions between the molecules in the cluster for every possible cluster. As a first approach to apply the model, they are generated using a force field-based molecular sampling algorithm which is developed in the following.

### Force Field Approach

The description of the energetic interactions of molecules in the presented sampling algorithm is achieved through the utilization of a molecular force field. The specific force field chosen for this work is the optimized potentials for liquid simulations force field in its all atom variant, in short OPLS-AA.^48^ It should be noted that, while the results presented in this work have been generated using the OPLS-AA force field, the general molecular sampling algorithm sketched out in this section can be used with other force field models or quantum mechanical calculations as well.

The choice to view molecules as rigid structures was made for reasons of simplicity and computational efficiency. This simplification reduces the need for calculating torsion, angle bending, and bond stretching transformations of molecules as well as the resulting changes in internal energy that these transformations cause.

As the aim of the model is to calculate excess properties, the only relevant part of the internal energy to be considered is the interaction energy. In terms of the OPLS-AA force field, this is the nonbonded intermolecular energy. It is represented by the summation of a Coulomb and a Lennard-Jones term for all pairs of atoms, where the atoms involved in the pairs must come from different molecules.^48^

In the all-atom (AA) approach, parameters are assigned to each atom in every molecule. These parameters can be determined manually by assigning appropriate groups to the individual molecules, based on a list of available groups for the atoms. An alternative to manual assignment is to use automated tools that handle the assignments. These have the benefit of generating more reproducible assignments since deterministic decision trees drive the process. The assignments used in this work are, whenever possible, based on the LigParGen web tool.^49^ The specific parameters used are given in the Supporting Information.

The calculation of the intermolecular energies requires, in addition to force field parameters, the distance between the atoms for each pair. To determine these, coordinates of the atom centers are required.

### Geometric States

The model section of this article introduces the states that a molecule can reside in. For each of the four sites in a cluster, a molecule of a specific component type is placed in one specific geometric state. The total number of possible clusters, *n*_Cl_, is given by
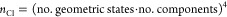
14

This work focuses on two-component mixtures considering 18 geometric states for each component type. According to eq 14, this results in a total number of (18·2)^4^ = 1,679,616 possible clusters.

As explained in the following, these 18 geometric states result from all possible combinations of six molecular orientation states and three distance states: The six molecular orientation states consider all possible combinations of three states for the inclination of an axis through a molecule and two states for the rotation angle around this axis; the three distance states account for the distances between the molecule pairs in the cluster.

### Molecular Orientation States

**Figure 4:**
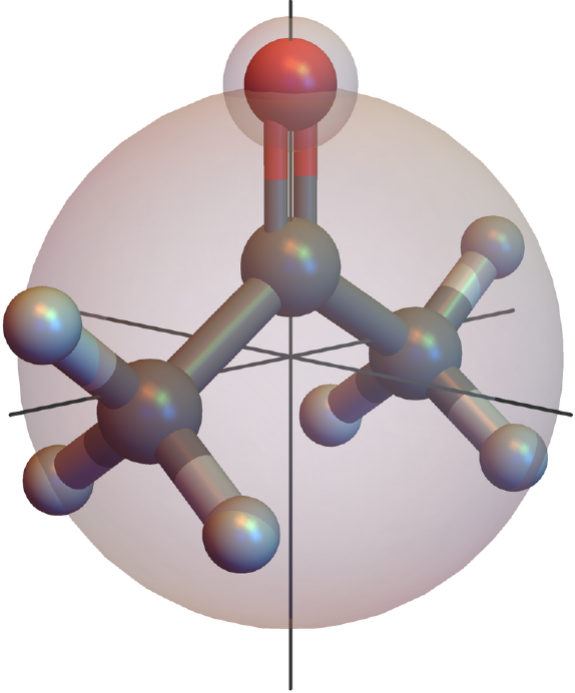
Acetone molecule, where the oxygen atom is chosen as the significant point on the unit sphere. The axis is spanned between the oxygen atom and the molecular center, i.e., the center of the sphere.

The molecular orientation is represented through an axis angle approach, where a significant point of the molecule is chosen, and the axis is spanned between this significant point and the molecule center. The coordinates of one of the atoms of the molecule determine the significant point, and its orientation in three-dimensional space is expressed by a point on a unit sphere. This is illustrated in Figure 4, using the example of an acetone molecule.

**Figure 5:**
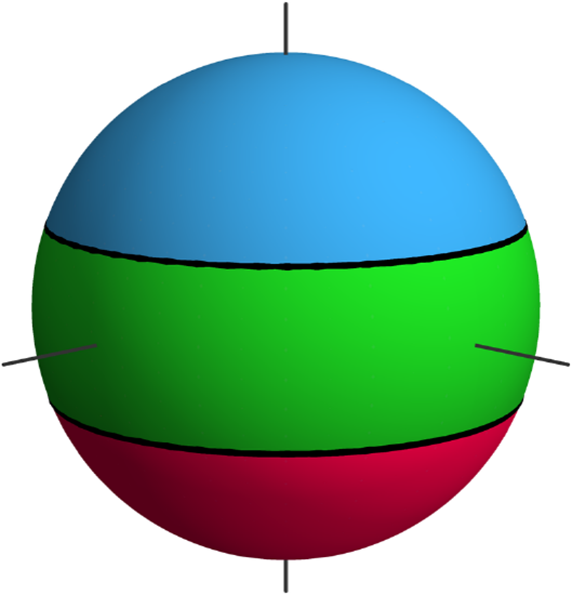
Surface segments of a sphere to divide the molecule orientation into three groups, based on the significant point belonging to one of these segments.

This sphere is now divided into three surface segments of equal area, and the first part of the molecular orientation states indicates the surface segment in which the significant point is located. The definition of the surface segments including their orientations in a cluster can be seen in Figure 5.

**Figure 6:**
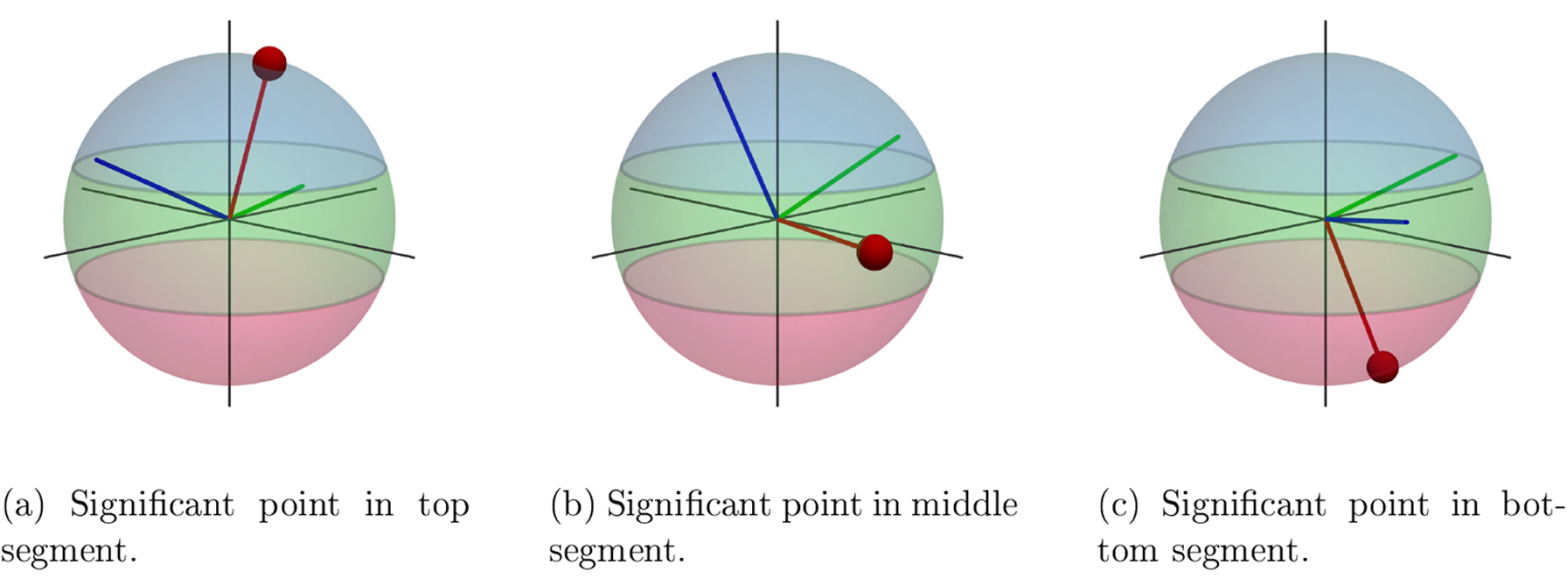
Random examples of significant points located in each of the three surface segments. Molecules are represented as a local coordinate system.

In a sampling step, one random point within each of these surface segments is chosen, and the molecule is oriented such that the axis spanned by the significant point goes through the random point, as shown in Figure 6.

**Figure 7:**
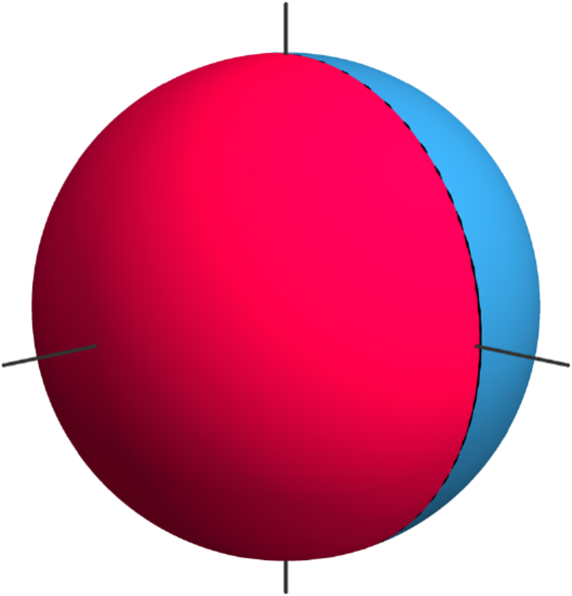
Illustration of the two rotational sections defining the second part of the molecular orientation states.

This completely defines the position of the significant point. However, there is still one degree of freedom remaining to fully define the orientation of the molecule. Consequently, the second part of the molecular orientation states considers two possibilities for the rotational angle around the axis through the significant point. For this purpose, the rotation around this axis is split into two sections of equal size. These describe all rotations within 180° to one side or the other of the original orientation, as shown in Figure 7. In a sampling step, a random angle from each section is chosen.

To summarize, the combination of the three states belonging to an area segment and the two states belonging to a rotational section completely defines the six molecular orientation states.

### Distance States

To completely define the geometric state of a cluster, the distances between its constituting molecules must be defined. To recall Figure 1, the molecule centers of neighboring molecules at sites ‘b’, ‘c’, and ‘d’ are placed on the axes of a Cartesian coordinate system originating at the center of the molecule placed on site ‘a’. In this work, the molecule center is defined as the arithmetic mean of the atom coordinates, as given in
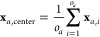
15using the example of molecule *A*.

There, **x**_*a*,center_ is the coordinate vector of the center of molecule *a*; vectors of atom coordinates are noted as **x**_*a*,*i*_, where *i* is the index of the atom in the molecule, and *o*_*a*_ is the number of atoms in molecule *a*.

This restriction of molecule centers to the axes, combined with fixed molecule orientations, reduces the remaining degrees of freedom to three. These three distance states are defined by belonging to one of three distance classes between the central molecule and each of its three neighbors.

**Figure 8:**
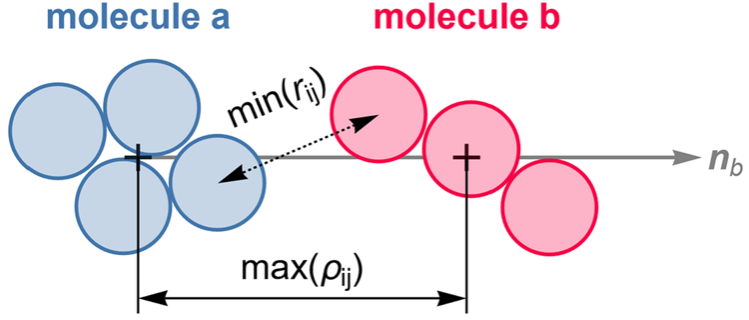
Simplified 2D schematic with pseudomolecules for illustrating the shift axis, **n**_b_, the molecular shift distance, ρ_*ij*_, and the distances between atoms, *r*_*ij*_.

Figure 8 illustrates how the intermolecular distance is determined, using the example of the molecule pair “ab”, formed by sites “a” and “b”, which is transferable one-to-one to pairs “ac” and “ad” as well.

Here, the index *i* refers to an atom of molecule a and index *j* to an atom of molecule b. The distance measure used between atoms *i* and *j*, *r*_*ij*_, is the Euclidean distance between the atom centers. Its definition is given in
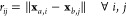
16

### Distance Shifting Algorithm

With these atom distances, it is now possible to formulate a distance shifting algorithm to determine the mutual distances of the molecules in the cluster. First, molecule *a* is placed at the origin. Second, molecule *b* is initially placed at the origin as well and then shifted outward along its respective shift axis, **n**_b_, until the smallest distance between any atom pair is greater than or equal to a minimal distance, *R*_min ,*ij*_. Put differently, the algorithm is searching for a shift distance, ρ_*ij*_, for which
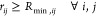
17holds for all atom pairs.

For this shift, Blanco^50^ and Fan et al.^51^ propose a method where a possible shift is determined for every atom pair using
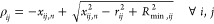
18

The newly introduced term *x*_*ij*,*n*_ is the coordinate difference along the shift axis, **n**_*B*_, as given in
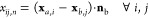
19

The maximum value of the list of generated shift distances for all atom pairs is applied to shift molecule *b* to its appropriate location. Last, the shifting algorithm requires a definition of the minimal distance, *R*_min ,*ij*_. Sweere and Fraaije^14^ proposed a possible definition of *R*_min ,*ij*_ for their excess Gibbs energy model PAC-MAC, which uses a pairwise molecular sampling algorithm with interaction energies also calculated from the OPLS-AA force field.
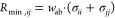
20

In eq 20, the minimal distance is expressed as the sum of the Lennard-Jones parameters σ_*ii*_ and σ_*jj*_, as indicators for atom sizes multiplied by a distance factor, *w*_ab_. With this definition, a distance factor can be defined for each molecule pair instead of each atom pair, since the atom size differences are taken into account.

**Figure 9:**
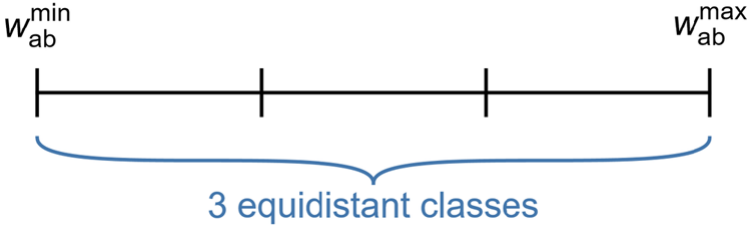
Illustration of the three distance classes and their bounds.

Finally, the three distance states are defined by equally dividing the range between a minimum and maximum value of *w*_ab_ into three classes, which is illustrated in Figure 9. These minimum and maximum values are input parameters that must be provided for each specific mixture.

To summarize, the combination of the three distance states and the six molecular orientation states explained before completely defines the 18 geometric states for each component type.

### Molecular Sampling Algorithm

The purpose of this algorithm is to provide a list of cluster energies as input to the thermodynamic model, eqs 13.

First of all, the entire sampling process starts by randomly selecting a numerical value for each state class, i.e., the six molecular orientation states and the three distance states, which is conducted for both component types separately. Combining these values results in 36 possible states for each cluster site. With these states as a basis, all possible clusters are constructed. In the course of this, the mean of the distance states of both cluster sites involved in an interaction pair is used to convert a molecule-based distance property into a pair based one.

Then, the shift distances are determined, and each neighbor is placed at its final position. Finally, the cluster interaction energy, *e*_*abcd*_^*ijkl*^, is determined for each cluster.

This entire process is composed of one individual sampling run. It is repeated multiple times with different random states sampled from each class. The averaged cluster energies of the different runs, a list of (18·2)^4^ = 1,679,616 values, represent the output of the molecular sampling algorithm and serve as input to the thermodynamic model, eqs 13.

For practical applications, the sampling algorithm has four parameters that need to be defined. These are the initial orientation of a molecule, the significant point on an enveloping sphere around a molecule, and the minimum and maximum distances of molecule pairs in the cluster.

## Results

### Boundary Cases of the Model

#### Model Consistency in the Limiting Case of Spherical Molecules

**Figure 10:**
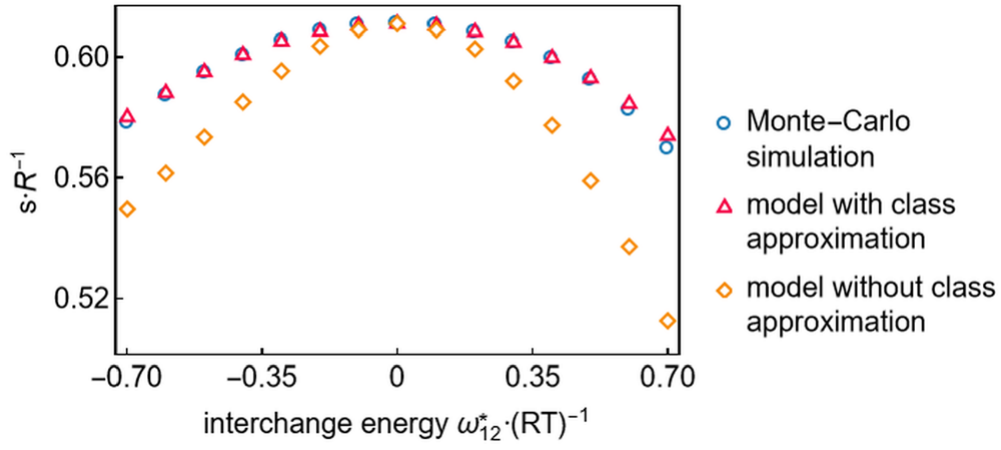
Entropy comparison for models using spherical molecules with Monte Carlo simulation data.^42^ Two versions of the same model are shown: One with the class approximation constraints, eq 8, and the other without. The interchange energy ω_12_^*^ ≡ ε_12_ + ε_21_ – ε_11_ – ε_22_ subsumes the interaction energies between spheres of different types, ε_12_ = ε_21_, and those of the same type, ε_11_ and ε_22_.

For spherical molecules with uniform interactions, it is not possible to distinguish between different orientations. If the distances between molecules are assumed to be equal, the only remaining distinguishable property of the molecules is the component type, and the probability distributions for cluster classes become trivial since each class has only one remaining member. The model then reduces perfectly to the system of equations of a previously published model developed for spherical molecules.^42^ This is illustrated in Figure 10 by plotting the dimensionless entropy over the dimensionless interchange energy that describes the energetic interactions between molecules of different component types.

Figure 10 also reveals the importance of introducing the class approximation constraints, eq 8, that significantly decrease model deviations from Monte Carlo simulation data.

#### Demixing

**Figure 11:**
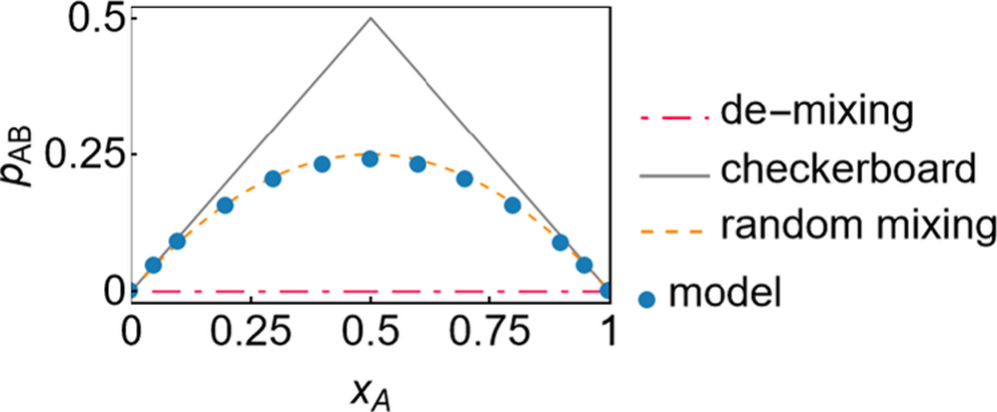
Boundary cases of the pair probability, *p*_*AB*_, over the global composition compared to model results for the mixture methane + ethane.

Demixing occurs in the case of overall strongly repulsive interactions between the component types, resulting in accumulations of molecules of the same type in the bulk of the mixture. In the present model, the pair probability, *p*_*AB*_, is a measure of how often molecules of different types are neighbors. Hence, the model reacts to the case of demixing by setting the value of *p*_*AB*_ to zero. Consequently, the probabilities of all clusters that contain a mixture of different component types must also be zero, and the only remaining clusters with nonzero probabilities are those solely involving pure component interactions. Figure 11 visualizes the boundary cases in terms of the pair probability, *p*_*AB*_.

The cases of demixing and a checkerboard like arrangement are the lower and upper limit for *p*_*AB*_. Model results typically are close to one of these special cases; most commonly, they are close to random mixing.

#### Checkerboard-like Arrangement

The opposite behavior to demixing occurs when there is a strong attraction between the different component types. The most extreme structure that a mixture in this case can form is one where molecules of the less common type are completely surrounded by molecules of only the other component type. For a mixture with a composition of *x*_*A*_ = 0.5, this results in a checkerboard like arrangement. This boundary case is indicated when *p*_*AB*_ = min(*x*_*A*_,*x*_*B*_).

#### Random Mixing

The limiting case of random mixing, representing an ideal mixture, exhibits special behavior since a continuous transition to this state is not possible for the free energy of the present model, which is because the Shannon function is formulated on the basis of *m* distinguishable states per component type. This prerequisite still holds when the energetic interactions get smaller right before random mixing is reached. In the case of random mixing, however, there are no more energetic interactions at all and, therefore, the only remaining distinguishable feature of a molecule is its component type. Hence, for the accurate description of the random mixing case within the model assumptions, *m* must equal one. The switch from *m* = 18 to *m* = 1 causes discontinuity in the entropy and therefore the free energy of the system. Consequently, to formally describe random mixing correctly, *m* = 1 must be set, which reduces the system of equations to that of a previously published model developed for spherical molecules.^42^ If the distinguishable states are not reduced to *m* = 1, then the entropy is not reduced to its random mixing value but takes on a higher value: For different values of *m*, the entropy of a random mixture increases by log  *m*. However, this addition of a constant term does not affect the probability distribution in equilibrium, and additionally, for the evaluation of excess properties, this term cancels out.

### Exemplary Application to Real Molecular Mixtures

As a proof of concept for the presented modeling approach, it is applied to four real molecular mixtures representing a variety of possible excess Gibbs-energy curves. As mentioned above, the interaction energies for these mixtures are determined using the OPLS-AA force field model.

The parameters required for applying the model to a specific mixture are the atom coordinates and the OPLS-AA force field parameters for the Coulomb and the Lennard-Jones terms which are generated using the LigParGen web tool.^49^ For the definition of the geometric states, the initial orientation and the significant atom, in addition to the minimum and maximum distance scaling factors, are further parameters. So far, these have to be adjusted individually for each investigated mixture. During the optimization of individual points, the temperature and global composition are also defined as parameters.

The mixtures can be classified by their associative behavior. Mixtures of methane + ethane and tetrahydrofuran + furan do not associate. Cross-association between the different component types without self-association occurs in the mixture chloroform + tetrahydrofuran. There, chloroform acts as a hydrogen donor, while tetrahydrofuran acts as a hydrogen acceptor. The remaining mixture forms both self- and cross-associations and is a system of isomers 1-propanol + 2-propanol. With this being a mixture in which both components are capable of self-association and cross-association, the four example systems are shown to represent a variety of association behaviors.

**Figure 12:**
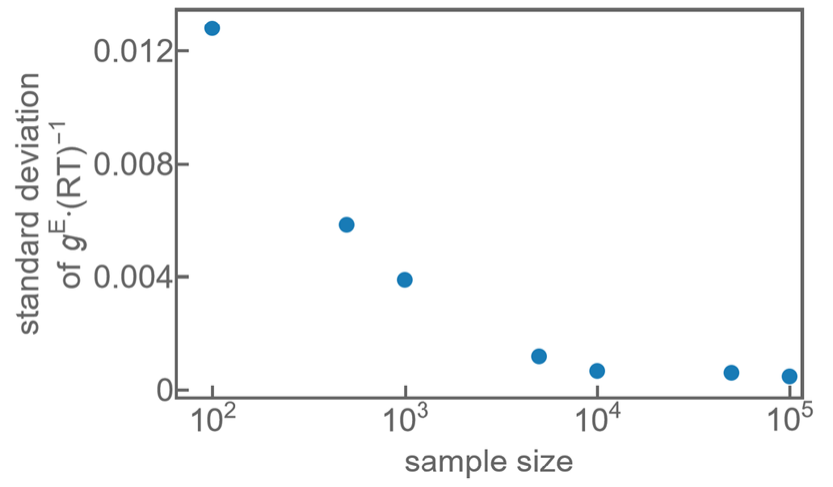
Standard deviation of dimensionless excess Gibbs-energy over the sample size, calculated from random sampling runs for the mixture of methane + ethane at a composition of *x*_*A*_ = 0.5.

The sampling section of this work introduces the fact that the energies of multiple individual sampling runs are averaged to generate the cluster energies that are provided to the model. The used sample size was chosen after assessing the deviations of excess Gibbs-energy between various sampling procedures that differed only in the initial values for the random number generator. This correlation is exemplarily shown for the mixture methane + ethane in Figure 12. It is recommended that the model is evaluated multiple times with different random number generator seed values for each investigated mixture to verify that the chosen sample size is appropriate for the specific interactions present in the mixture. All results in this work were generated using a sample size of 10^4^.

**Figure 13:**
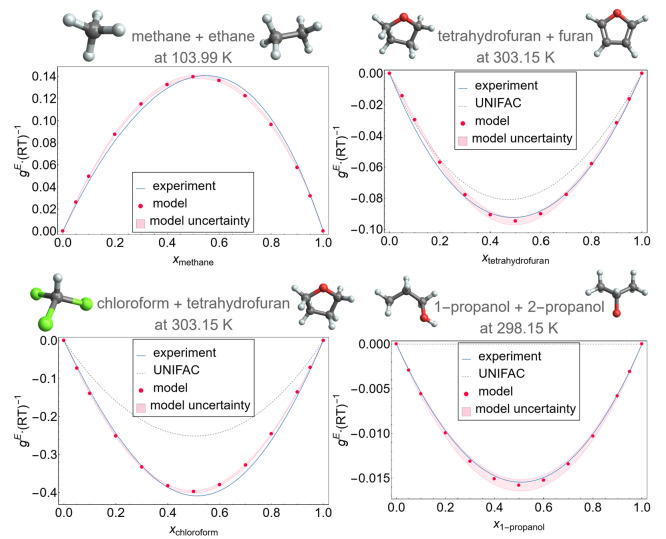
Excess Gibbs-energy model results of six mixtures at constant temperatures with model uncertainties calculated as the standard deviation between evaluations that differ in pseudo-random-number generator seed values for the sampling algorithm. The results are compared to experimental data for methane + ethane from Gomes de Azevedo and Calado,^52^ for tetrahydrofuran + furan and chloroform + tetrahydrofuran from Byer et al.,^53^ and 1-propanol + 2-propanol from Haase and Tillmann.^54^ When available, the results are also compared to the UNIFAC model using the 2021 parameter set of the UNIFAC Consortium.^55^ Experimental data from which the experiment curves have been derived are provided in the Supporting Information, Figures S5–S7.

The four parameters to be defined for the sampling algorithm comprise the initial orientation of a molecule, the significant point on an enveloping sphere around a molecule, and the minimum and maximum distances of molecule pairs in the cluster. The initial orientations were arbitrarily chosen. The significant points that were found to have a low impact on the results were specified individually per component, choosing one of the outer atoms of a molecule. The minimum and maximum distances were found by a comprehensive screening of possible combinations for each binary system and comparison of the resulting *g*^E^-curves with experimental data, resulting in [0.16, 0.66] for methane + ethane, [0.12, 0.62] for tetrahydrofuran + furan, [0.18, 0.56] for chloroform + tetrahydrofuran, and [0.37, 0.92] for 1-propanol +2-propanol. The resulting isothermal excess Gibbs-energy curves of the four binary systems are given in Figure 13.

It can be seen that the model can achieve results with small deviations from experimental data, especially when compared to predictions from the UNIFAC model. For the four example mixtures the model results in *g*^*E*^-curves that are slightly more symmetric than the experimental data.

## Summary and Conclusion

A thermodynamic model has been developed that uses clusters of molecules to describe the cooperative effects in a binary condensed phase mixture in equilibrium. It is based on the use of informational Shannon entropy as a synonym for thermodynamic entropy to describe a sequential construction of the mixture in terms of discrete Markov-chains. First results for four real molecular mixtures show that this model is able to accurately describe the mixture’s experimental excess Gibbs-energy given that the appropriate cluster energies are provided, which in the current implementation is achieved with a molecular sampling algorithm similar to that of the PAC-MAC method.

Through the model, it is demonstrated that Shannon entropy can be a useful starting point for thermodynamic modeling, since even simple model assumptions lead to promising agreement with experimental data and the model can also distinguish structural isomers, which is not possible with many established approaches for the excess Gibbs-energy of molecular mixtures. Consequently, the proposed modeling approach offers a variety of research opportunities. First and foremost, the molecular sampling algorithm needs to be further developed to ensure that its parameters, especially the distance classes, are uniformly applicable to systems of the same chemical group, which is in the direction of developing a predictive model. Second, as each component type is assigned the same number of states, disregarding different complexities of the components, the model could be extended to support these asymmetric complexities. Another possible extension would be to use a different distribution function and different coordination numbers, with the latter having an influence on the overall shape of the cluster.

## Supporting Information Available

Thermodynamic consistency check; used molecule parameters; initial value strategies for numeric evaluations; experimental data for comparison (PDF)
