# Supplementary material for: Discrete Modeling Approach for Cluster-Based Excess Gibbs-Energy of Molecular Liquids
Source: Ind Eng Chem Res. 2023 Nov 7;62(46):20017–28. doi: 10.1021/acs.iecr.3c03084 (PMC10682997; doi:10.1021/acs.iecr.3c03084)
Supplement: Supplementary file 1 — ie3c03084_si_001.pdf [file ie3c03084_si_001.pdf]

Discrete Modeling Approach for Cluster-based Excess Gibbs-energy of Molecular Liquids Supporting Information

Christoph Mayer and Thomas Wallek*

Institute of Chemical Engineering and Environmental Technology, Graz University of Technology, Graz 8010, Austria

E-mail: thomas.wallek@tugraz.at

Phone: +43 (0)316 6902 7966

Thermodynamic consistency

The thermodynamic consistency is proven using the area test. The resulting deviations are within machine precision.

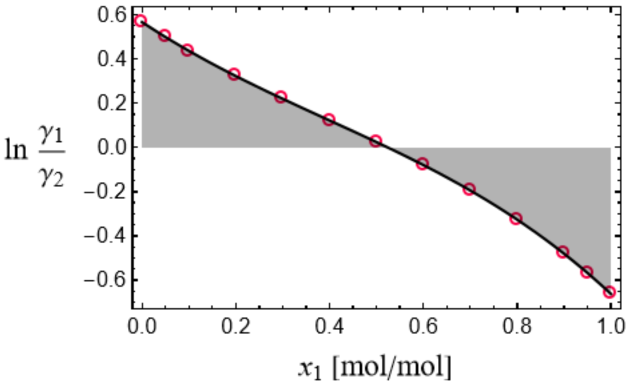

Figure S1: Area test for the mixture methane + ethane.

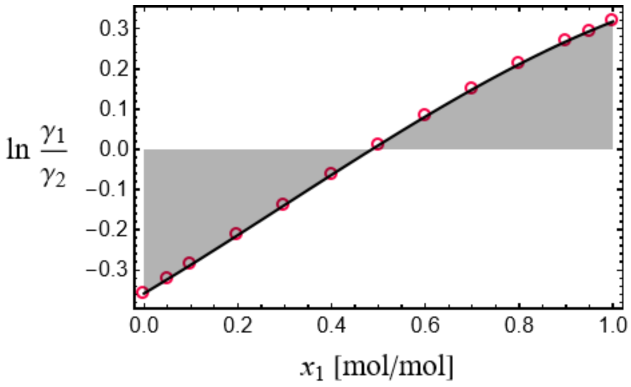

Figure S2: Area test for the mixture tetrahydrofuran + furan.

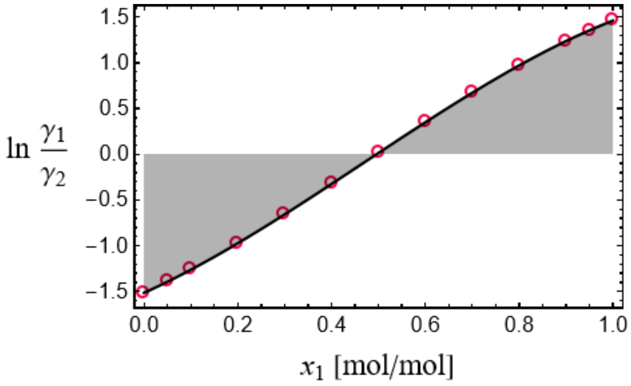

Figure S3: Area test for the mixture chloroform + tetrahydrofuran.

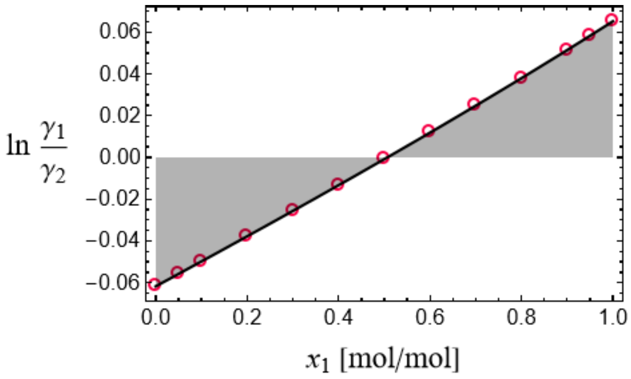

Figure S4: Area test for the mixture 1-propanol + 2-propanol.

Molecule parameters

This section contains the necessary parameters to reproduce the results presented in the accompanying article. Atom coordinates, x, y, z, and force-field parameters were generated using the LigParGen online tool using the $1.14 \cdot CM1A$ setting.[1]

Chloroform

atom type	x [Å]	y [Å]	z [Å]	q [e$^-$]	σ [Å]	ϵ [kcal $\cdot$ mol^{-1}]
C	-0.03868	-0.03902	-0.1023	0.0793	3.5	0.066
Cl	-0.64548	-1.11242	1.17	-0.0873	3.4	0.3
Cl	-0.64548	1.60898	0.1321	-0.0873	3.4	0.3
Cl	1.73272	-0.05132	-0.1346	-0.0873	3.4	0.3
H*	-0.40308	-0.40622	-1.0652	0.1826	2.5	0.03

*. . . significant atom

Ethane

atom type	x [Å]	y [Å]	z [Å]	q [e$^-$]	σ [Å]	ϵ [kcal $\cdot$ mol^{-1}]
C*	-0.756	1.39e-17	0.	-0.2396	3.5	0.066
C	0.756	1.39e-17	0.	-0.2396	3.5	0.066
H	-1.1404	0.6586	0.7845	0.0799	2.5	0.03
H	-1.1404	0.3501	-0.9626	0.0799	2.5	0.03
H	-1.1405	-1.0087	0.1781	0.0799	2.5	0.03
H	1.1404	-0.3501	0.9626	0.0799	2.5	0.03
H	1.1405	1.0087	-0.1781	0.0799	2.5	0.03
H	1.1404	-0.6586	-0.7845	0.0799	2.5	0.03

Furan

atom type	x [Å]	y [Å]	z [Å]	q [e$^-$]	σ [Å]	ϵ [kcal $\cdot$ mol^{-1}]
C	0.787789	0.661211	0.0059	-0.2276	3.55	0.07
C	-0.507711	1.12361	-0.0048	-0.0934	3.55	0.07
O*	-1.38221	0.0843111	-0.0099	-0.1586	2.9	0.14
C	-0.640511	-1.05349	-0.0037	-0.0928	3.55	0.07
C	0.701489	-0.752089	0.0062	-0.2268	3.55	0.07
H	1.68139	1.26891	0.012	0.1839	2.42	0.03
H	-0.952811	2.10871	-0.0101	0.2161	2.42	0.03
H	-1.20211	-1.97719	-0.008	0.2155	2.42	0.03
H	1.51469	-1.46399	0.0124	0.1838	2.42	0.03

Methane

atom type	x [Å]	y [Å]	z [Å]	q [e$^-$]	σ [Å]	ϵ [kcal $\cdot$ mol^{-1}]
C	0.00002	-0.00002	0.	-0.2994	3.5	0.066
H*	0.55412	0.79958	0.4965	0.0748	2.5	0.03
H	0.68332	-0.81342	-0.2536	0.0748	2.5	0.03
H	-0.77818	-0.37352	0.6692	0.0748	2.5	0.03
H	-0.45928	0.38738	-0.9121	0.0748	2.5	0.03

1-propanol

atom type	x [Å]	y [Å]	z [Å]	q [e$^-$]	σ [Å]	ϵ [kcal $\cdot$ mol^{-1}]
C	-1.40648	0.503292	-0.280358	-0.2359	3.5	0.066
C	0.111117	0.476392	-0.263558	-0.226	3.5	0.066
C	0.645917	-0.678308	0.571242	0.0102	3.5	0.066
O*	2.06712	-0.662908	0.561542	-0.5884	3.12	0.17
H	-1.76478	1.34179	-0.885458	0.0842	2.5	0.03
H	-1.80808	0.618292	0.731542	0.0842	2.5	0.03
H	-1.80998	-0.420408	-0.707258	0.0842	2.5	0.03
H	0.496917	1.42389	0.130642	0.0958	2.5	0.03
H	0.492317	0.399292	-1.28876	0.0958	2.5	0.03
H	0.306717	-1.64351	0.181342	0.0951	2.5	0.03
H	0.314817	-0.593408	1.61094	0.0951	2.5	0.03
H	2.35442	-0.764408	-0.361858	0.4055	0.	0.

2-propanol

atom type	x [Å]	y [Å]	z [Å]	q [e^-]	σ [Å]	ϵ [$\mathrm{kcal \cdot mol^{-1}}$]
C	-1.21502	0.0131417	-0.499408	-0.282	3.5	0.066
C	0.305583	0.0220417	-0.470408	0.0689	3.5	0.066
C	0.840983	0.527742	0.860292	-0.2819	3.5	0.066
O*	0.787783	-1.29806	-0.699908	-0.5872	3.12	0.17
H	-1.62052	1.01844	-0.349908	0.0912	2.5	0.03
H	-1.62002	-0.646858	0.275692	0.0912	2.5	0.03
H	-1.57632	-0.370958	-1.45961	0.0912	2.5	0.03
H	0.683183	0.654542	-1.28071	0.1293	2.5	0.03
H	0.512683	-0.113058	1.68619	0.0912	2.5	0.03
H	0.508883	1.55144	1.05839	0.0912	2.5	0.03
H	1.93618	0.508242	0.863392	0.0912	2.5	0.03
H	0.456583	-1.86666	0.0159917	0.4058	0.	0.

Tetrahydrofuran

atom type	x [Å]	y [Å]	z [Å]	q [e$^-$]	σ [Å]	ϵ [kcal · mol^{-1}]
C	-0.752077	0.774292	0.0501615	-0.2197	3.5	0.066
C	-0.784477	-0.736008	0.0255615	-0.2197	3.5	0.066
C	0.666823	-1.16281	-0.000838462	0.0005	3.5	0.066
O*	1.43322	-0.0268077	0.408962	-0.3829	2.9	0.14
C	0.717123	1.13309	-0.0216385	0.0004	3.5	0.066
H	-1.18368	1.13949	0.989062	0.1075	2.5	0.03
H	-1.32298	1.22879	-0.765138	0.1075	2.5	0.03
H	-1.33578	-1.13451	-0.834138	0.108	2.5	0.03
H	-1.27958	-1.11751	0.926062	0.108	2.5	0.03
H	0.978623	-1.44241	-1.01264	0.0979	2.5	0.03
H	0.871223	-2.00321	0.668362	0.0979	2.5	0.03
H	1.01792	1.36449	-1.05004	0.0974	2.5	0.03
H	0.973623	1.98309	0.616262	0.0974	2.5	0.03

Initial value strategies

To ensure quick and stable numerical results, the following strategies for initial values have proven suitable. First, both pure component systems are evaluated, because there the system of equations is reduced to one free variable, which is the distribution parameter of the respective pure component cluster class, α_{AAAA} and α_{BBBB}. For these calculations multiple initial values are used representing boundary cases of the variables and a value in between. They are $(10^{-6}, 0.5, 1)$.

Next, the results of the pure components are used to determine initial values for the mixture. For the pair probability, p_{AB}, the value for the case of random mixing is used, as

seen in eq 1. The initial values of the distribution parameters are given in eqs 2.

$$p_{AB}^{IV} = x_A(1 - x_A) \tag{1}$$

$$\begin{aligned}
\alpha_{AAAA}^{IV} &= \alpha_{AAAA}^{pure} && (2a)\\
\alpha_{AAAB}^{IV} = \alpha_{AABA}^{IV} = \alpha_{ABAA}^{IV} = \alpha_{BAAA}^{IV} &= \frac{3\alpha_{AAAA}^{pure} + \alpha_{BBBB}^{pure}}{4} && (2b)\\
\alpha_{AABB}^{IV} = \alpha_{ABAB}^{IV} = \alpha_{ABBA}^{IV} &= \frac{\alpha_{AAAA}^{pure} + \alpha_{BBBB}^{pure}}{2} && (2c)\\
\alpha_{BAAB}^{IV} = \alpha_{BABA}^{IV} = \alpha_{BBAA}^{IV} &= \frac{\alpha_{AAAA}^{pure} + \alpha_{BBBB}^{pure}}{2} && (2d)\\
\alpha_{ABBB}^{IV} = \alpha_{BABB}^{IV} = \alpha_{BBAB}^{IV} = \alpha_{BBBA}^{IV} &= \frac{\alpha_{AAAA}^{pure} + 3\alpha_{BBBB}^{pure}}{4} && (2e)\\
\alpha_{BBBB}^{IV} &= \alpha_{BBBB}^{pure} && (2f)
\end{aligned}$$

Experimental data used in Figure 13

The ‘experiment’ lines in Figure 13 were determined mostly from fitting $\{T, P, x_i, y_i\}$ or $\{T, P, x_i\}$ datasets to the Redlich-Kister equation; for the mixture tetrahydrofuran + furan, only Redlich-Kister parameters were available in literature.

For the three mixtures where data points were available, the following Figures show experimental points together with the ‘experiment’ lines of Figure 13. Data for methane + ethane are taken from Gomes de Azevedo and Calado[2], for tetrahydrofuran + furan and chloroform + tetrahydrofuran from Byer et al.[3], and 1-propanol + 2-propanol from Haase and Tillmann[4]. For the mixture chloroform + tetrahydrofuran, the $\{T, P, x_i, y_i\}$ dataset from Paul[5] was used to generate the data points to which the smooth line taken from the previous publication is compared.

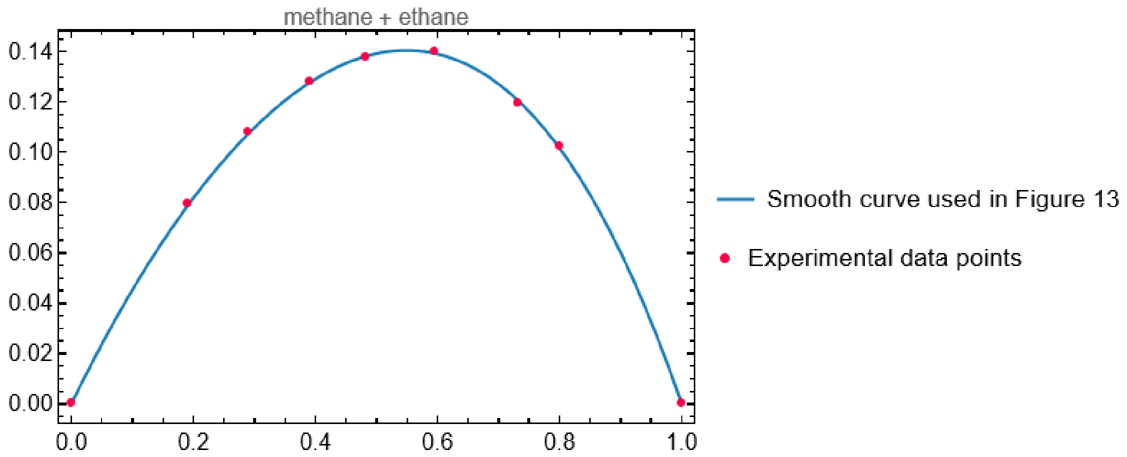

Figure S5: Comparison of data points and Redlich-Kister polynomials for the mixture methane + ethane.

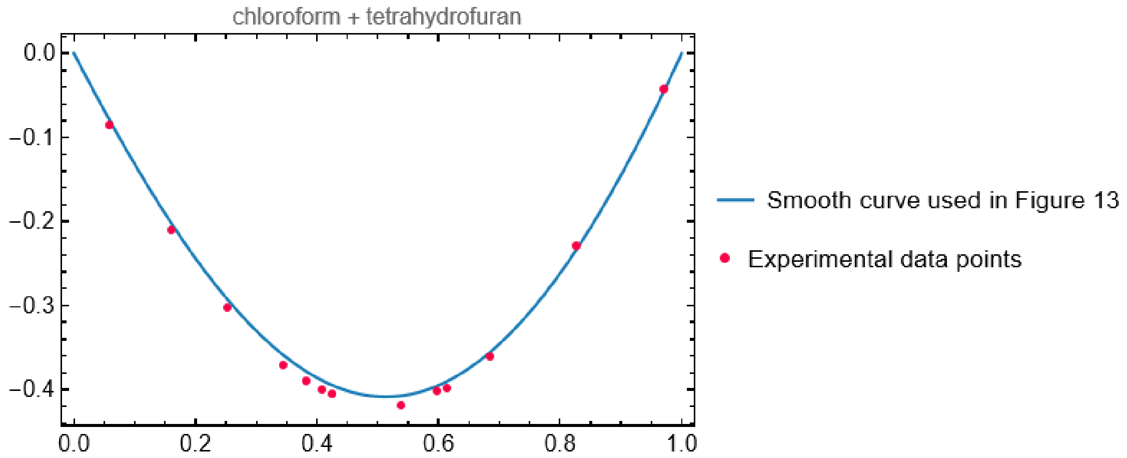

Figure S6: Comparison of data points and Redlich-Kister polynomials for the mixture chloroform + tetrahydrofuran.

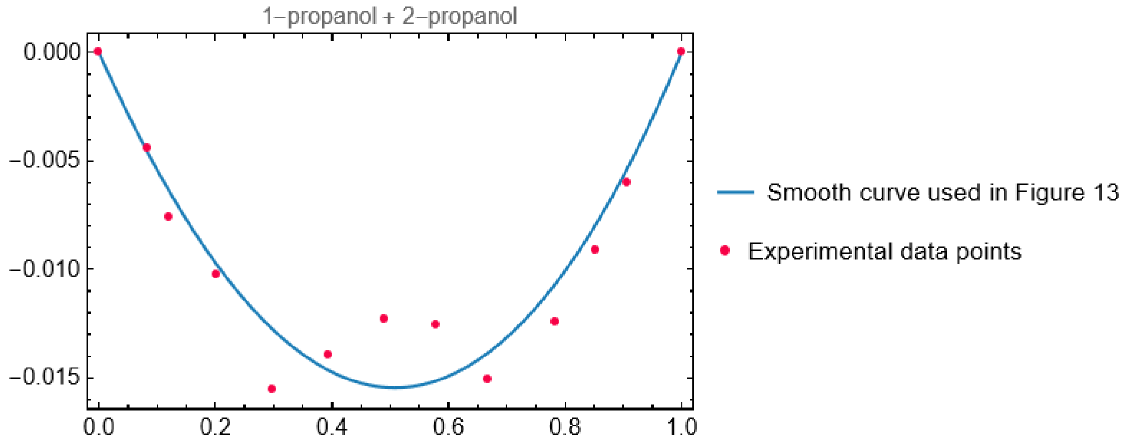

Figure S7: Comparison of data points and Redlich-Kister polynomials for the mixture 1-propanol + 2-propanol.

References

(1) Dodda, L. S.; Cabeza de Vaca, I.; Tirado-Rives, J.; Jorgensen, W. L. LigParGen web server: an automatic OPLS-AA parameter generator for organic ligands. *Nucleic Acids Research* **2017**, *45*, W331–W336.

(2) Gomes de Azevedo, E.; Calado, J. Thermodynamics of liquid methane+ethane. *Fluid Phase Equilibria* **1989**, *49*, 21–34.

(3) Byer, S. M.; Gibbs, R. E.; Van Ness, H. C. Vapor-liquid equilibrium: Part II. Correlations from P-x data for 15 systems. *AIChE Journal* **1973**, *19*, 245–251.

(4) Haase, R.; Tillmann, W. Mixing Properties of the Liquid Systems Methanol + 2-Propanol and 1-Propanol + 2-Propanol. *Zeitschrift für Physikalische Chemie* **1995**, *192*, 121 – 131.

(5) Paul, H.-I. Experimentelle Untersuchung der Flüssigkeits-Dampf-Phasengleichgewichte und volumetrischen Eigenschaften binärer und ternärer Mischungen. *VDI Forschungsh. Reihe 3 Verfahrenst. 135* **1987**, 1.
